# Systematic Analysis and Accurate Identification of DNA N4-Methylcytosine Sites by Deep Learning

**DOI:** 10.3389/fmicb.2022.843425

**Published:** 2022-03-15

**Authors:** Lezheng Yu, Yonglin Zhang, Li Xue, Fengjuan Liu, Qi Chen, Jiesi Luo, Runyu Jing

**Affiliations:** ^1^School of Chemistry and Materials Science, Guizhou Education University, Guiyang, China; ^2^Department of Pharmacology, School of Pharmacy, Southwest Medical University, Luzhou, China; ^3^School of Public Health, Southwest Medical University, Luzhou, China; ^4^School of Geography and Resources, Guizhou Education University, Guiyang, China; ^5^Department of Endocrinology and Metabolism, The Affiliated Hospital of Southwest Medical University, Luzhou, China; ^6^Department of Pharmacy, The Affiliated Hospital of Southwest Medical University, Luzhou, China; ^7^School of Cyber Science and Engineering, Sichuan University, Chengdu, China

**Keywords:** deep learning, convolutional neural network, recurrent neural networks, one-hot encoding, attention mechanism, DNA N^4^-methylcytosine

## Abstract

DNA N^4^-methylcytosine (4mC) is a pivotal epigenetic modification that plays an essential role in DNA replication, repair, expression and differentiation. To gain insight into the biological functions of 4mC, it is critical to identify their modification sites in the genomics. Recently, deep learning has become increasingly popular in recent years and frequently employed for the 4mC site identification. However, a systematic analysis of how to build predictive models using deep learning techniques is still lacking. In this work, we first summarized all existing deep learning-based predictors and systematically analyzed their models, features and datasets, etc. Then, using a typical standard dataset with three species (*A. thaliana*, *C. elegans*, and *D. melanogaster*), we assessed the contribution of different model architectures, encoding methods and the attention mechanism in establishing a deep learning-based model for the 4mC site prediction. After a series of optimizations, convolutional-recurrent neural network architecture using the one-hot encoding and attention mechanism achieved the best overall prediction performance. Extensive comparison experiments were conducted based on the same dataset. This work will be helpful for researchers who would like to build the 4mC prediction models using deep learning in the future.

## Introduction

DNA methylation, one of the most important epigenetic modifications in many organisms, plays an important role in a vast number of cellular processes (Rathi et al., 2018, p. 4). According to the location where a methylated group occurs in the DNA sequence, there are many types of DNA methylation. For example, the most common types are N^6^-methyladenine (6mA), C^5^-Methylcytosine (5mC), and N^4^-methylcytosine (4mC), which are found in both eukaryotic and prokaryotic genomes (Davis et al., 2013; Jeltsch and Jurkowska, 2014; Blow et al., 2016). The first discovered 5mC is the most studied type of methylation in eukaryotes, and it plays crucial roles in a broad range of biological processes, including gene expression, imprinting, regulation and transposon suppression (Suzuki and Bird, 2008), and is even involved in various diseases, such as neurological disorders, cancers and diabetes (Jones, 2012; Yao and Jin, 2014). In prokaryotes, 6mA and 4mC constitute the majority of DNA base methylations, and they are frequently used to distinguish between benign host DNA and potentially exogenous pathogenic DNA (Heyn and Esteller, 2015). The former is essential for regulating gene expression, genomic imprinting, DNA mismatch repair and cell developments (Xiao et al., 2018; Hasan et al., 2021), while the latter is critical for the regulation of DNA replication, repair, expression, and differentiation (Cheng, 1995; Chen et al., 2016), and can even prevent the enzymatic degradation of host DNA (Schweizer, 2008).

Compared to the studies on 5mC and 6mA, progress on 4mC has been relatively slow due to the lack of effective detection methods. There are several experimental methodologies, such as mass spectrometry, methylation-precise PCR, single-molecule real-time sequencing (SMRT), and 4mC-Tet-assisted bisulfite-sequencing (4mCTABseq), have been efficiently used to detect the epigenetic sites (Buryanov and Shevchuk, 2005; Flusberg et al., 2010; Doherty and Couldrey, 2014; Yu et al., 2015). However, these approaches are commonly regarded as expensive, time-consuming and complex, and not suitable for high throughput assays at the whole genome level. Therefore, there is a strong incentive to develop alternative approaches to support experimental efforts properly. The computational approaches could be used to effectively and accurately identify 4mC sites based on machine learning (ML) algorithms and genomic sequences.

Since iDNA4mC was established as the first 4mC predictor in 2017, at least 33 predictors have been developed to date, of which 24 predictors have been built in the last 2 years (Supplementary Table 1). These predictors typically make use of machine learning algorithms to learn from available data to perform novel predictions and gain new insights. Recently, a variety of machine learning algorithms are useful for this goal, such as support vector machine (SVM) (Chen et al., 2017; He et al., 2019; Wei et al., 2019a,b; Lv et al., 2020b; Zhao et al., 2020), random forest (RF) (Hasan et al., 2020a,b; Lv et al., 2020a; Alghamdi et al., 2021; Zulfiqar et al., 2021a), Markov model (MM) (Yang et al., 2020), and the combined or ensemble methods (Gong and Fan, 2019; Manavalan et al., 2019a,b; Tang et al., 2020; Li et al., 2021), extreme gradient boosting (XGBoost) (Wang et al., 2021) and Laplacian Regularized Sparse Representation (Ding et al., 2021). As shown in Supplementary Table 1, SVM is the most widely used traditional machine learning algorithms in the model development and method comparison for 4mC prediction, followed by RF. Moreover, two excellent overviews of computational predictions and applications for DNA 4mC are available from Manavalan et al. (2020) and Xu et al. (2021), and some predictors that can identify multiple epigenetic modifications have been developed, such as iDNA-MT (Yang et al., 2021) and ZayyuNet (Abbas et al., 2021a). Although some methods based on traditional machine learning algorithms have achieved very good performance in predicting 4mC sites, they still depend heavily on manually crafted features and fail to automatically learn intrinsic features from raw DNA sequences. In addition, all these traditional methods may require complicated pretreatments or consume too much time, especially when dealing with large data sets or a large number of features. Thus, there is still room for improvement in the ML-based prediction of DNA 4mC, chiefly in the areas of the speed and accuracy.

Deep learning algorithms avoid the need to manually craft informative features and instead automatically learn features of DNA sequences through the iterative aggregation of features in each layer of the network (Jing et al., 2020; Yu et al., 2020, 2021). So far, at least 14 predictors have been reported to be developed by the deep learning algorithms (Table 1). Among the various types of deep networks that have been proposed, convolutional and convolutional-based neural networks (CNNs and CNN-) are the most frequently applied types of neural network for modeling the DNA 4mC sites. The strength of CNN is that its initial convolution layer corresponds to motif detectors, where position weight matrices (PWMs) are not hard-coded, but solely learned from data. Despite CNNs are showing great promise for DNA 4mC site analysis, there are many challenges that remain to be addressed. For example, why is it better to use CNNs and their variants to develop the predictive models instead of other types of neural networks? Current applications in identifying the 4mC sites have been limited to a specific deep learning type, such as the CNNs, however, few comprehensive comparison studies have been performed to evaluate the relative performance of different deep learning methods regarding available DNA sequencing datasets of different sizes, technologies, species, and complexity. In addition, the deep neural networks continue to be treated mostly as black-box function approximators, as it is unclear from the model itself why a given classification is made. More specifically, currently available deep learning tools offer little to explanation or visibility for why specific features are selected over others during training, or which nucleotides in both upstream and downstream the 4mC sites have the greatest effect on the predictive performance, or why a specific motif in the input sequence is selected over others. Therefore, it is essential and urgent to make a comprehensive, integrated and visual analysis of deep learning algorithms on the prediction of DNA 4mC sites.

**TABLE 1:** Summary of 14 existing deep learning-based prediction tools.

Year	Tools	Architectures	Evaluation strategy	References
2019	4mCCNN	CNN	10-fold CV	Khanal et al., 2019
2020	4mcDeep-CBI	CNN-BiLSTM	Threefold CV	Zeng et al., 2020
	Deep4mcPred	ResNet, RNN, attention mechanism	10-fold CV	Zeng and Liao, 2020
	Deep4mC	CNN, attention mechanism	n-fold CV (*n* = 4, 6, 8, 10)	Xu et al., 2021
	DeepTorrent	CNN-RNN, attention mechanism	10-fold CV	Liu et al., 2021
	DNC4mC-Deep	CNN	10-fold CV	Wahab et al., 2020
2021	4mCNLP-Deep	CNN	*k*-fold CV (*k* = 3, 5, 10)	Wahab et al., 2021
	4mCPred-CNN	CNN	10-fold CV	Abbas et al., 2021b
	4mC-w2vec	CNN	Fivefold CV	Khanal et al., 2021
	iRG-4mC	CNN	10-fold CV	Lim et al., 2021
	4mCPred-MTL	Multi-task learning coupled with transformer, attention mechanism	10-fold CV	Zeng et al., 2021
	i4mC-Deep	CNN	10-fold CV	Alam et al., 2021
	Deep-4mCW2V	CNN	10-fold CV	Zulfiqar et al., 2021b
	DCNN-4mC	CNN	10-fold CV	Rehman et al., 2021

*CNN, convolutional neural network; BiLSTM, bidirectional long short-term memory; ResNet, Residual Network; RNN, recurrent neural network; CNN-RNN, convolutional neural network and recurrent neural network.*

In this article, based on a comprehensive overview of existing deep learning-based 4mC prediction methods (Table 1 and Supplementary Table 1), we provide a valuable reference for many issues to consider when designing, implementing, and analyzing the deep learning models for DNA 4mC sites. This work provides three key contributions. First, we evaluate different types of deep learning models and sequence encoding methods for DNA 4mC sites and present results on *A. thaliana*, *C. elegans*, and *D. melanogaster* datasets using standard performance metrics. To our knowledge, this is the first comprehensive benchmarking of deep learning algorithms and implementations with large-scale 4mC datasets. Second, we provide original implementations of deep learning models and utility functions for training, evaluation, interpretation and visualization. Finally, we offer practical guidance for selecting the combination of model architecture and encoding methods best suited to the scientific question addressed for end-users and tool developers.

## Materials and Methods

**FIGURE 1:**
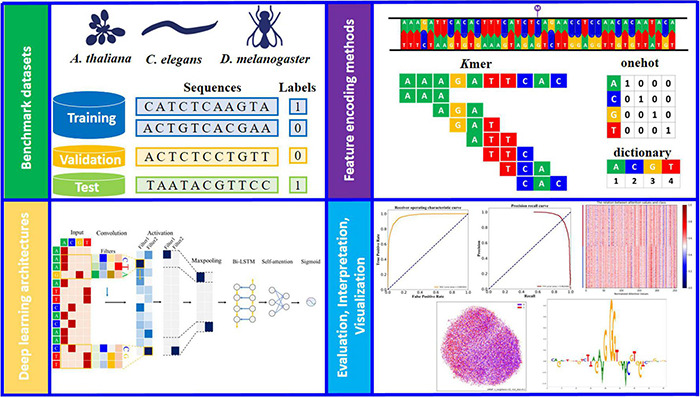
Schematic overview of the study design (see detailed descriptions and notations in section “Materials and Methods”). Three model organism (*A. thaliana*, *C. elegans*, and *D. melanogaster*) are used to generate the benchmark datasets. Each dataset is balanced between the positive and negative samples and randomly split into training, validation and test sets. For input sequence, the channel axis encodes different colors (such as green, blue, orange and red), for one-hot and dictionary encoded sequences (A: [1, 0, 0, 0] for one-hot; A: 1 for dictionary, and so on). The appropriate deep learning architecture is designed and trained on the basis of benchmarking studies. Further evaluation and interpretation of the proposed model *via* a variety of visual analysis methods.

As mentioned above, many software tools have recently been developed to classify 4mC data and identify genomic loci using the deep learning techniques. This diversity of the methods and tools presents its own challenge. Alternative views on a problem can produce multiple distinct tools to achieve the same general purpose. However, small differences in method selection or model design in these tools can impact the classification accuracy, and even produce inconsistent results. The fast pace of biotechnology and computer science makes it increasingly difficult to choose a right deep learning method or tool for accurate analysis and interpretation of the data at hand. Moreover, it is important to understand how these different tools, referred to as deep learning classifiers, work and how to determine the best method for a given sample type, model organism, or application. Here, we introduced the core principles of 4mC sequence classification methods, described how to design, train, implement and evaluate the classifiers, and used these approaches to benchmark several commonly used deep learning algorithms. To combine the strengths of these approaches, we designed a series of experiments using a standard dataset of DNA 4mC loci from three different species, as well as different deep learning architectures and feature encoding methods where possible. To account for dataset and algorithms differences, we further compared the performance of these algorithms on a uniform dataset. Based on benchmarking results, we devised the DeepDNA4mC, which leverages the hybrid CNN-RNN architecture and self-attention mechanism to learn precise motif representations of significant information about diverse biological sequences. Finally, we provided recommendations for their use and described future directions for the expansion of this field. An overview of the experimental design and data analysis is presented in Figure 1 and following sections.

### Benchmark Datasets

Some well-known databases of DNA 4mC loci have been established, such as MethSMRT (Ye et al., 2016), DNAmod (Sood et al., 2019), and MDR (Liu et al., 2019). Several standard datasets were generated from sequences mined from the above-mentioned databases, which have been used to develop more than 20 prediction tools so far (as shown in Supplementary Table 1). An increasing number of species are involved in this process, including *Arabidopsis thaliana* (*A. thaliana*), *Caenorhabditis elegans* (*C. elegans*), *Casuarina equisetifolia* (*C. equisetifolia*), *Drosophila melanogaster* (*D. melanogaster*), *Escherichia coli* (*E. coli*), *Fragaria vesca* (*F. vesca*), *Geoalkalibacter subterraneus* (*G. subterraneus*), *Geobacter pickeringii* (*G. pickeringii*), *Mus musculus* (*M. musculus*), *Rosa chinensis* (*R. chinensis*), *Saccharomyces cerevisiae* (*S. cerevisiae*), *Tolypocladium* sp. *SUP5-1* (*Ts. SUP5-1*), and even their cross-species (Wahab et al., 2020). Recently, Manavalan et al. (2020) reviewed the 4mC site prediction methods for eight species and further evaluated their predictive performance using their self-constructed datasets.

Chen et al. (2017) provided a golden standard dataset, which consists of DNA sequences from six species. This dataset was adopted in 12 subsequent studies for model performance assessment and comparison. Unfortunately, with the rapid growth of 4mC data, the relatively small sample size of Chen’s dataset is a clear drawback to developing effective predictors, especially for the deep learning methods. Hence, a larger dataset established by Zeng and Liao (2020) was selected as our first benchmark dataset, which is called the Zeng_*2020_1*. This dataset was generated by merging three different subsets of species: *A. thaliana*, *C. elegans*, and *D*. *melanogaster*. All positive samples in this dataset were extracted from the MethSMRT database (Ye et al., 2016) and all sequences were all 41 base pairs in length with methylated cytosine centrally located. To avoid redundancy and homology bias, all DNA sequences were aligned by CD-HIT with an 80% identity threshold (Fu et al., 2012). For each species, 20,000 positive samples and 20,000 negative samples were randomly selected to balance the potential confounding factors (Supplementary Table 2). The negative samples were not identified by the SMRT sequencing technology, but they were also 41 bp in length and the center of the sequence was a non-methylated cytosine.

To further evaluate our model performance across data sets, another benchmark dataset established by Zeng et al. (2020) (named Zeng_*2020_2*) was also employed in this work. This dataset consists of two parts, one of which is the existing data of *C. elegans* provided by Ye et al. (2016), and the other part is composed of many new data extracted from the updated MethSMRT database. All samples in this dataset have a sequence length of 41 bp, and they were also processed by CD-HIT to remove the redundant DNA sequences. In particular, the samples with the Modification QV (modQV) score higher than 30 have been removed during data processing. Ultimately, 11,173 positive samples and 6,635 negative samples from *C. elegans* formed the second benchmark dataset. The reason for choosing above benchmark data is that two exiting deep learning predictors, 4mcDeep-CBI (Zeng et al., 2020) and 4mCNLP-Deep (Wahab et al., 2021), were developed based on this dataset, in addition to the golden standard dataset obtained from the study of Chen et al. (2017) All datasets described herein can be freely downloaded from https://github.com/jingry/autoBioSeqpy/tree/2.0/examples/DeepDNA4mC/data.

### Feature Encoding Methods

In contrast to traditional machine learning methods, the deep learning algorithms can automatically extract valuable features from data, which does not require feature engineering. Even so, the string of nucleotide characters (A, C, G, T) have to be transformed into a matrix format before being input into the neural network layer. We considered two commonly proposed methods for representing DNA sequences based on deep learning techniques: one-hot and dictionary encoding methods (Zhou and Troyanskaya, 2015; Veltri et al., 2018). For the one-hot encoded nucleotides, A, C, G, and T are encoded as [1, 0, 0, 0], [0, 1, 0, 0], [0, 0, 1, 0], and [0, 0, 0, 1], respectively. Therefore, each sequence is represented as a *N* × 4 matrix, where *N* is the length of the input DNA sequence. While for the latter encoding method, the sequence is transformed as an *N*-dimensional vector, i.e., where A is encoded as the number 1, C is encoded as the number 2, G is encoded as the number 3, and T is encoded as the number 4. In addition, we applied the *k*-mer mechanism (*k* = 2, 3) for both encoding methods to represent the input sequences. For instance, a trinucleotide is a *k*-mer for which *k* = 3.

### Deep Learning Architectures

Here, we selected three representative deep learning algorithms constructed with different network architectures, including a convolutional neural network (CNN); a recurrent neural network (RNN) with bidirectional long short-term memory cells (BiLSTM); and a hybrid convolutional-recurrent neural network (CNN-RNN) (LeCun et al., 2015; Yu et al., 2020). More details of these network architectures are described below.

Generally, the ordinary CNN constructure is composed of few convolutional layers, pooling layers adjected by the convolutional layer and fully connected layers before final activate layer. In the convolutional layer, various filters are applied to scan through input sequences, so that the network can capture short range correlations from local regions rather than the whole. Several operations (e.g., max, average, and sum) are used in the pooling layer to effectively reduce variance and increase translational invariance from local features extracted from the previous convolutional layer. The ReLU function and dropout method are usually adopted to prevent the vanishing gradient problem and overfitting. For the CNN, the principal network architecture included convolution with 150 filters of size 5 to transform the one-hot encoded DNA sequence followed by ReLU and width two maximum pooling. The flattened pooling output was then passed to a fully connected layer of 256 hidden nodes with 0.5 × dropout, which finally connected to a sigmoid activation node that outputs a probability of the input sequence as a 4mC site.

The architecture for RNN mainly consisted of stacked bidirectional LSTM cells (Bi-LSTM), which can capture long range sequence dependencies. Bi-LSTM can process sequences in both directions, forward and backward directions, and thus often captures the context better. The Bi-LSTM layer with 128 hidden neurons and one layer of depth gave the best performance. A dropout rate of 0.2 was applied to the Bi-LSTM layer to prevent overfitting by avoiding co-adaptation between the hidden neurons. The output layer also contained sigmoid activation and one neuron representing the probability of the sequence of interest being a 4mC site.

The CNN-RNN architecture were made up of four types of layers: a convolutional layer, a pooling layer, a recurrent layer and a self-attention layer. The sequentially convolutional and pooling layers were usually constructed at the first step, and the recurrent and self-attention layers were used in the latter phase. Using convolution layers before the recurrent layer is a way for absorbing more information from the neighbor of the base in the sequence and thus enhance learning ability of the recurrent layers. In other words, the CNN-RNN architecture can take the local environment of the base into RNN layers for learning. More precisely, the architecture first incorporated one 1D convolutional layer with 150 filters of width three along with a 1D max-pooling layer with size = 2. The output from these layers was then inputted into a Bi-LSTM layer with 128 neurons. To help the recurrent layer more attention to specific sequence patterns, a self-attention layer was adopted in the latter phase, following the Bi-LSTM layer. Using self-attention layer allows the model learns more information not limited by the sequential of the residues, and thus can increase stability of the model. The dropout rate was 0.2 for different layers. The activation of the output layer was also a sigmoid function.

Binary cross-entropy loss between the target and predicted outputs was minimized using Adam optimizer during training. While training, 10% of the training samples were used as a validation set for monitoring validation loss. We stopped training when validation loss had not improved for 20 epochs, and we took the model parameters that had achieved that minimum validation loss forward as the final model. We performed a grid search to exhaustively test hyperparameter combinations of number of convolution filters (50, 150, 250), convolution filter lengths (3, 5, 7, 9, 11), pooling sizes (2, 4, 6, 8, 10), number of neurons in the Bi-LSTM layer (16, 32, 64, 128, 256), dropout probability between all layers (0.2, 0.3, 0.4, 0.5), mini batches of size (16, 32, 64, 128), and learning rate (0.001, 0.005, 0.01, 0.05, 0.1). Best performance was achieved with a learning rate of 0.01; batch size of 64, and other hyperparameter combinations were shown in the following section. The numbers of the parameters in the models depends on the used structures and the number of channels. For example, the convolution layer with 128 channels and 9 kernel length contains about 300,000 trainable parameters, and a Bi-LSTM layer with 128 input size and 256 hidden size contains about 240,000 parameters. Therefore, the final model which contains both CNN and Bi-LSTM layers contains more than 540,000 parameters in this work. We randomly divided the sequences contained in each class of our dataset into a training set and a test set, with the training set containing about 80% of the samples and the test set 20%. For each architecture, we repeated the dataset division and the training procedure 10 times and obtained 10 trained models. The average of their outputs was used as the predicted output. In addition, 10-fold nested cross-validation was employed to train the all models using onefold for testing, and the nine remaining folds for training. All code was written in Python 3.7 and all neural network development was done using the autoBioSeqpy software (Jing et al., 2020) with the Tensorflow GPU backend to enable parallel calculation of gradient. For general user applications, the CPU backend is also sufficient. The computing environment for this work is a CentOS workstation with a Xeon E5-2620 CPU and an RTX 2080Ti GPU.

### Evaluation, Interpretation, and Visualization

To quantitatively evaluate the class-wise prediction quality, we calculate the recall (*Sensitivity*), precision (*PRE*), accuracy (*ACC*), *F*-value, and Matthew’s correlation coefficient (*MCC*) as comparison metrics, which are defined in Supplementary Table 3. Several of these metrics can also be compared visually to capture the tradeoff between true-positive rate (sensitivity) and false-positive rate (1—specificity) using the receiver operating characteristic (ROC) curves or precision-recall (PR) curves (Rifaioglu et al., 2019). These visual metrics can also be used to measure classification and prediction performance using a single value, such as the area under the ROC or PR curve.

To help understand what sequence features were learned and make the “black box” deep learning model more transparent, we generated attention weights heat maps to highlight the hidden neurons that most contribute to the predicted classes from the output for the recurrent layers. For each hidden neuron, the self-attention layer is calculated to compare with other vectors in the sequence and obtain the attention weights of the neuron to adjust the values. The following equation shows the additive attention mechanism:


(1) $$v=softmax\left(\tanh\left(X\cdot W_{q}+X\cdot W_{k}+\varepsilon_{bh}\right)\cdot W_{a}+\varepsilon_{ab}\right)\cdot X$$

Where the ‘*W_q_*,’ ‘*W_k_*,’ and ‘*W_a_*’ are weights which could be updated during the epochs, ‘ε_*bh*_’ and ‘ε_*ab*_’ are two bias vectors. As a brief description, the attention layer can calculate the similarity between vectors and modify the weights according to the similarity.

To understand the evolution of the data in each hidden layer of the model, we visualized the sample representations of network architecture in two dimensions. We used the output of a certain hidden layer as the extracted output features, which were then projected into a 2D manifold *via* uniform manifold approximation and projection (UMAP) (McInnes et al., 2020) with parameter values: n_neighbors:15, min-dist: 0.1. Next, we used a two-color scheme to refer to 4mC loci/locus (red) and non-4mC loci/locus (purple).

To measure input feature importance for predicting 4mC sites, the Deep SHAP method was used. We extracted one-hot encoding features and trained the deep learning models with their best hyperparameter configurations determined from the optimization strategy as described above. In the Deep SHAP method, a per-sample importance score (SHAP value) is assigned to each feature value in the one-hot matrix from the trained deep learning models. The SHAP value is computed based on the game theory:


(2) $$\phi_{i}\left(f,x\right)=\sum_{S\subseteq S_{all/\left\{i\right\}}}\frac{\left|S\right|!\left(M-\left|S\right|-1\right)!}{M!}\left[f_{x}\left(S\cup \left\{i\right\}\right)-f_{x}\left(S\right)\right]=\sum_{S\subseteq S_{all/\left\{i\right\}}}\frac{1}{C_{M}^{\left|S\right|}\left(M-\left|S\right|\right)}\left[f_{x}\left(S\cup \left\{i\right\}\right)-f_{x}\left(S\right)\right]$$

Where *M* is the number of features, *S* is a subset of the features, *f* is the model, *S*_*all*_/{*i*} is all the possible subset exclude feature *i*, and *f_x_* is the conditional expectation function. With the SHAP values, a model can be represented as a linear combination of the SHAP values:


(3) $$f\left(x\right)=\phi_{0}\left(f\right)+\sum_{i=1}^{M}\phi_{i}\left(f,x\right)$$

Where ϕ_0_(*f*) = *f*_*x*_(∅). We finally show SHAP value distributions for the entire dataset using the average absolute value to give an overview of feature importance in the deep learning models. The SHAP values consists an importance matrix whose shape is similar with the position frequency matrix, but SHAP values scoring the contribution of the inputs instead of recording the frequency. If the predicting performance of the built model is satisfactory, the observed patterns from SHAP values will contain potential value for further exploring.

## Results and Discussion

We evaluated the performance of different deep learning methods on two standard datasets (Zeng_2020*_1* and Zeng_*2020_2*). Details of datasets are provided in section “Materials and Methods.” Briefly, these datasets cover three representative model organisms that include *A. thaliana*, *C. elegans*, and *D. melanogaster*. In addition, both datasets considered balance and unbalanced sample designs. In each dataset, we investigated the effect of two different feature encoding methods (one-hot and dictionary encodings) and three different deep learning architectures (CNN, RNN, and CNN-RNN), as well as a variety of hyperparameters (filter numbers, kernel and pooling sizes, and so on) on the prediction of 4mC sites. In particular, we presented several innovative approaches to visualize and understand the deep learning models. We seek to characterize the model’s black box behavior, with trying to elucidate its inner working mechanism and shed light on its internal representations. We focus on interpreting the inputs and outputs of the model and explaining its predictions.

### Performance Assessment on the *Zeng_2020_1* Dataset

#### Choosing the Number of Filters, Kernel and Pool Sizes in Convolutional Neural Networks and Convolutional-Recurrent Neural Networks for *Caenorhabditis elegans*

We proposed the first benchmark to assess the performance of two deep learning architectures, systematically varying their tunable hyperparameters on a grid of values, on the *C. elegans* dataset with known C^4^-methylcytosine (4mC) identity. We ran a total of 260 experiments to assess the influence of parameter changes on the performance of each architecture. Specifically, for each convolutional layer, we varied the number of filters to be either 50, 150, or 250, and we varied the widths of filters to be either 3, 5, 7, 9, or 11. The benchmark results based on the five comparison metrics were summarized in Table 2. It is clear that the best prediction results were obtained for both architectures when the number of filters was set to 150. Here, the CNN obtained the highest scores of *ACC* (88.5%), *F*-value (88.6%), *PRE* (87.7%), and *MCC* (0.770) using 150 filters, and the CNN-RNN also obtained the best values for the four metrics (88.7, 88.9, 87.8, and 0.775) using the same filters. For the kernel size, the CNN obtained the best results when the kernel size was set to 7. As the kernel size increased, the *MCC* value first increased and then decreased, reaching a peak at width 7. While the best performance of CNN-RNN was achieved when the kernel size was 3, its *ACC* and *MCC* values decreased as the kernel size increased. After the best performing filter number and kernel size were determined, we found that both architectures achieved their best performance with width two maximum pooling. For example, with pooling size of 2, the *MCC* of CNN and CNN-RNN were 0.770 and 0.775, respectively. It is worth noting that the overall prediction performance of CNN-RNN architecture outperforms that of the CNN for all three hyperparameters.

**TABLE 2:** Hyperparameter optimization of CNN and CNN-RNN architectures on the *C. elegans* dataset.

#Dim	Architecture	ACC (%)	*F*-value (%)	Recall (%)	PRE (%)	MCC
**Filter number**						
50		88.4	88.6	90.3	87.4	0.770
**150**	CNN	**88.5**	**88.6**	**89.6**	**87.7**	**0.770**
250		87.9	88.1	89.3	86.9	0.759
50		88.3	88.5	90.5	86.6	0.766
**150**	CNN-RNN	**88.7**	**88.9**	**90.0**	**87.8**	**0.775**
250		88.4	88.5	89.4	87.6	0.767
**Kernel size**						
3		87.9	88.1	89.2	87.0	0.759
5		88.5	88.6	89.6	87.7	0.770
**7**	CNN	**88.5**	**88.5**	**89.2**	**87.9**	**0.771**
9		88.1	88.3	89.2	87.4	0.763
11		87.8	88.1	89.7	86.5	0.757
3		**89.0**	**89.4**	**92.6**	**86.5**	**0.783**
5		88.7	88.9	90.0	87.8	0.775
**7**	CNN-RNN	88.2	88.5	90.9	86.2	0.765
9		88.1	88.3	89.6	87.1	0.764
11		87.7	87.9	89.6	86.3	0.754
**Pooling size**						
**2**		**88.5**	**88.6**	**89.6**	**87.7**	**0.770**
4		87.6	87.7	88.5	86.9	0.752
6	CNN	86.6	86.6	86.7	87.0	0.732
8		84.8	85.0	85.9	84.0	0.696
10		81.5	81.7	82.5	81.1	0.631
**2**		**88.7**	**88.9**	**90.0**	**87.8**	**0.775**
4		88.7	87.5	87.6	87.5	0.751
6	CNN-RNN	86.6	86.8	88.0	85.7	0.733
8		78.0	78.0	77.0	79.1	0.560
10		76.7	76.5	76.3	76.9	0.534

*The bold values highlight the best parameters of the deep learning methods such as CNN and RNN.*

#### Choosing the Long Short-Term Memory Cells Size in Recurrent Neural Networks and Convolutional-Recurrent Neural Networks for *Caenorhabditis elegans*

As one of the most important parameters of the recurrent neural network (Zhang et al., 2020), the performance of LSTM size (also known as the number of hidden cells) in RNN and CNN-RNN architectures was also assessed by the same *C. elegans* dataset. In this process, we varied the number of hidden cells from 16, 32, 64, 128, to 256 to examine their influence on the prediction performance (Table 3). Clearly, the best performance of the RNN and CNN-RNN architectures was obtained using 128 hidden cells. The RNN achieved an average *ACC*, *F*-value, *Recall*, and *MCC* of 89.3, 89.7, 93.1, and 0.788%, respectively, which were slightly higher than those of CNN-RNN (89.0, 89.4, 92.6, and 0.783%). Furthermore, the *ACC* and *MCC* values of RNN were found to increase with the increasing LSTM size, with the highest values being recorded at 128 hidden cells, however, the performance of the CNN-RNN did not show significant change.

**TABLE 3:** LSTM sizes optimization of RNN and CNN-RNN architectures on the *C. elegans* dataset.

#Dim	Architecture	ACC (%)	*F*-value (%)	Recall (%)	PRE (%)	MCC
16		80.1	80.1	80.1	80.3	0.604
32		85.1	84.8	83.9	86.2	0.705
64	RNN	88.1	87.9	87.0	89.0	0.762
128		**89.3**	**89.7**	**93.1**	**86.5**	**0.788**
256		88.9	88.9	88.8	89.0	0.778
16		87.8	88.3	91.8	85.1	0.760
32		88.9	88.8	88.5	89.3	0.778
64	CNN-RNN	88.8	88.9	89.5	88.3	0.777
128		**89.0**	**89.4**	**92.6**	**86.5**	**0.783**
256		88.9	88.9	89.5	88.4	0.778

*The bold values highlight the best parameters of the deep learning methods such as CNN and RNN.*

### Performance Comparison of Different Deep Learning Architectures

We benchmarked the performance of three different deep learning models (CNN, RNN, and CNN-RNN) across three representative model organisms: *A. thaliana*, *C. elegans*, and *D. melanogaster* for intra-dataset evaluation. As a benchmark, we optimized hyperparameters for each model, then the top-performing tuned models were evaluated for a fair comparison. A summary of the experimental results is provided in Table 4. CNN, the most commonly used deep learning architecture for the 4mC prediction, performed worse performance for all three species, achieving an overall *MCC* of 0.673, 0.782, and 0.720 for *A. thaliana*, *C. elegans*, and *D. melanogaster*, respectively. The CNN-RNN provided the highest *MCC* score for *D. melanogaster* (0.736) and the second highest *MCC* scores (0.678 and 0.783) for *A. thaliana* and *C. elegans*, respectively. The RNN achieved the best performance for *A. thaliana* and *C. elegans*, with *MCC* scores of 0.701 and 0.788, respectively.

**TABLE 4:** Performance comparison of different deep learning models on the *A. thaliana*, *C. elegans*, and *D. melanogaster* datasets.

Model	ACC (%)	*F*-value (%)	Recall (%)	PRE (%)	MCC
***A. thaliana***					
CNN	83.6	83.7	84.2	83.3	0.673
**RNN**	**85.0**	**85.1**	**86.6**	**83.7**	**0.701**
CNN-RNN	83.9	84.3	86.1	83.9	0.678
***C. elegans***					
CNN	89.1	89.3	90.8	87.8	0.782
**RNN**	**89.3**	**89.7**	**93.1**	**86.5**	**0.788**
CNN-RNN	89.0	89.4	92.3	86.5	0.783
***D. melanogaster***					
CNN	86.0	86.1	87.2	85.1	0.720
RNN	85.9	85.8	87.1	84.8	0.720
**CNN-RNN**	**86.8**	**87.0**	**88.7**	**85.3**	**0.736**

*The bold values highlight the best methods of the species (i.e., A. thaliana, C. elegans, and D. melanogaster).*

### Incorporating the Self-Attention Mechanism Improves Model Performance

In the RNN or CNN-RNN models, relatively important features can be effectively captured by introducing a self-attention mechanism. This remarkably complex mechanism has been widely used for classification tasks in various fields (Hu et al., 2019; Liang et al., 2021; Tian et al., 2021), including the prediction of 4mC sites (Zeng and Liao, 2020; Liu et al., 2021; Xu et al., 2021; Zeng et al., 2021). To test whether the attention mechanism improves the predictive performance, we implemented the self-attention layer in different network architectures of RNN and CNN-RNN. We found that the CNN-RNN architecture with the attention mechanism (CNN-RNN_attention) achieved the best prediction performance for *C. elegans* and *D. melanogaster*, showing *ACC* scores of 89.4 and 87.4%, *PRE* scores of 88.6 and 86.0%, and *MCC* scores of 0.789 and 0.749, respectively (Table 5). The classification performance increased from CNN-RNN to CNN-RNN_attention from 89.0% accuracy to 89.4% accuracy for *C. elegans*, and from 86.8% accuracy to 87.4% accuracy for *D. melanogaster*. The accuracy remained almost unchanged for *A. thaliana*. These results suggest that incorporating self-attention mechanism into these models may improve performance of 4mC site prediction. In summary, we chose the CNN-RNN architecture with the attention mechanism as the final model to develop a new deep learning method called DeepDNA4mC, and further compared it with other exiting methods.

**TABLE 5:** Performance comparison of RNN and CNN-RNN architectures with or without the attention mechanism.

Species	Models	ACC (%)	*F*-value (%)	Recall (%)	PRE (%)	MCC
*A. thaliana*	RNN	85.0	85.1	86.6	83.7	0.701
	RNN_Attention	84.6	85.1	87.1	93.2	0.692
	CNN-RNN	83.9	84.3	86.1	83.9	0.678
	CNN-RNN_Attention	**83.9**	**83.8**	**84.0**	**83.7**	**0.678**
*C. elegans*	RNN	89.3	89.7	93.1	86.5	0.788
	RNN_Attention	89.3	89.5	91.4	87.6	0.788
	CNN-RNN	89.0	89.4	92.3	86.5	0.783
	CNN-RNN_Attention	**89.4**	**89.4**	**90.3**	**88.6**	**0.789**
*D. melanogaster*	RNN	85.9	85.8	87.1	84.8	0.720
	RNN_Attention	87.0	87.4	89.6	85.3	0.742
	CNN-RNN	86.8	87.0	88.7	85.3	0.736
	CNN-RNN_Attention	**87.4**	**87.7**	**89.6**	**86.0**	**0.749**

*The bold values highlight the best methods of the species (i.e., A. thaliana, C. elegans, and D. melanogaster).*

### Performance Comparison of Different Encoding Methods for Convolutional-Recurrent Neural Network_Attention Model

To investigate the effect of different encoding methods on model performance, we trained CNN-RNN_attention model on the three representative model organisms. The five metrics representing performance are summarized in Table 6. As expected, the 1-mer_onehot outperformed other encoding methods in terms of accuracy and *MCC*. That is, the average accuracy and *MCC* values obtained from applying this method were (83.9%, 0.678) for *A. thaliana*, (89.4%, 0.789) for *C. elegans*, and (87.4% and 0.749) for *D. melanogaster*, respectively. In addition, we found that the performance of the model gradually decreased as the *K*-value increased, regardless of the encoding method used. The downward trend was more obvious for one-hot encoding, with *AAC* and *MCC* values decreasing from 1 to 3-mer by 2.0% and 0.039 for *A. thaliana*, 1.5% and 0.032 for *C. elegans*, and 2.8% and 0.055 for *D. melanogaster*, respectively. A more moderate downward trend was observed for the dictionary encoding, with just decreasing by 0.9% and 0.018 for *A. thaliana*, 0.5% and 0.011 for *C. elegans*, and 0.5% and 0.012 for *D. melanogaster*.

**TABLE 6:** Performance comparison of the CNN-RNN_attention model with different encoding methods.

Species	coding	ACC (%)	*F*-value (%)	Recall (%)	PRE (%)	MCC
*A. thaliana*	1-mer_onehot	**83.9**	**83.8**	**84.0**	**83.7**	**0.678**
	2-mer_onehot	83.3	83.5	84.8	82.5	0.668
	3-mer_onehot	81.9	82.0	82.9	81.3	0.639
	1-mer_dict	83.8	84.1	85.5	82.8	0.678
	2-mer_dict	83.4	83.7	85.3	82.3	0.670
	3-mer_dict	82.9	83.0	82.5	83.5	0.660
*C. elegans*	1-mer_onehot	**89.4**	**89.4**	**90.3**	**88.6**	**0.789**
	2-mer_onehot	88.8	89.0	90.5	87.6	0.777
	3-mer_onehot	87.9	87.9	88.3	87.5	0.757
	1-mer_dict	88.5	88.7	90.6	87	0.772
	2-mer_dict	88.3	88.4	89.7	87.2	0.766
	3-mer_dict	88.0	88.1	88.6	87.8	0.761
*D. melanogaster*	1-mer_onehot	**87.4**	**87.7**	**89.6**	**86.0**	**0.749**
	2-mer_onehot	86.3	86.3	86.8	85.9	0.727
	3-mer_onehot	84.6	84.7	85.0	84.5	0.694
	1-mer_dict	86.6	86.8	87.6	86.2	0.734
	2-mer_dict	86.7	86.8	87.2	86.4	0.734
	3-mer_dict	86.1	86.3	87.7	85.1	0.722

*The bold values highlight the best methods of the species (i.e., A. thaliana, C. elegans, and D. melanogaster).*

### Comparison of DeepDNA4mC With Other State-of-the-Art Methods

To further evaluate the performance of our developed CNN-RNN_attention model with one-hot encoding (DeepDNA4mC), we compared it with other five state-of-the-art methods, including iDNA4mc (Chen et al., 2017), 4mcPred (He et al., 2019), 4mcPred_SVM (Wei et al., 2019a), 4mcPred_IFL (Wei et al., 2019b), and Deep4mcPred (Zeng and Liao, 2020). The five methods used the same standard dataset (Zeng*_2020_1* dataset) and 10-fold cross-validation for evaluation, which makes the comparison possible. We evaluated *ACC*, *Recall* (also called Sensitivity), and *MCC* for each method, and their results are shown in Table 7. It is noteworthy that all results of the five state-of-the-art methods come directly from the study of Zeng and Liao (2020). Clearly, DeepDNA4mC had the best overall performance for *C. elegans* and *D. melanogaster*, and the second-best overall performance for *A. thaliana*. DeepDNA4mC was capable of accurately predicting 4mC sites with average *MCC* of 0.678, 0.789, and 0.749, respectively. We found that deep learning-based predictors, DeepDNA4mC and Deep4mcPred, both obtained satisfactory results, indicating the great potential of deep learning in DNA 4mC site prediction.

**TABLE 7:** Performance comparison of DeepDNA4mC and other five exiting predictors.

Species	Predictors	ACC (%)	Recall (%)	*MCC*
*A. thaliana*	iDNA4mc	76.1	76.6	0.520
	4mcPred	76.8	75.5	0.536
	4mcPred_SVM	78.7	77.8	0.573
	4mcPred_IFL	82.2	80.3	0.644
	Deep4mcPred	84.4	86.0	0.689
	DeepDNA4mC	**83.9**	**84.0**	**0.678**
*C. elegans*	iDNA4mc	78.0	79.0	0.560
	4mcPred	82.6	82.5	0.652
	4mcPred_SVM	81.5	82.4	0.631
	4mcPred_IFL	88.0	89.0	0.761
	Deep4mcPred	89.3	91.5	0.787
	DeepDNA4mC	**89.4**	**90.3**	**0.789**
*D. melanogaster*	iDNA4mc	81.2	83.3	0.620
	4mcPred	82.2	82.4	0.646
	4mcPred_SVM	83.0	83.8	0.661
	4mcPred_IFL	87.3	86.5	0.745
	Deep4mcPred	87.1	87.6	0.742
	DeepDNA4mC	**87.4**	**89.6**	**0.749**

*The bold values highlight the best methods of the species (i.e., A. thaliana, C. elegans, and D. melanogaster).*

### Evaluation, Interpretation and Visualization of the DeepDNA4mC

**FIGURE 2:**
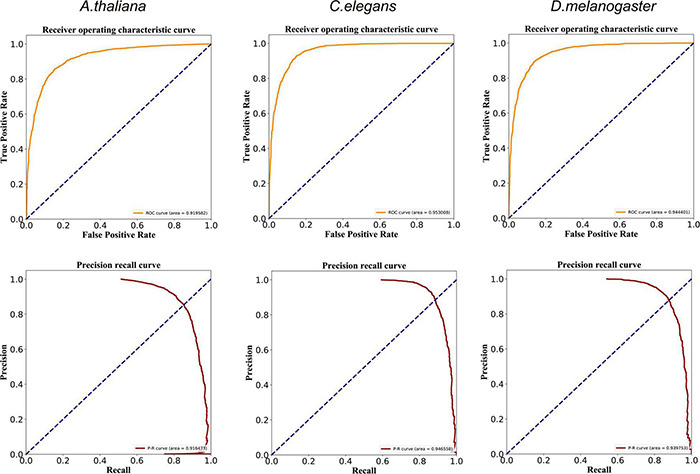
ROC and PR curves of the DeepDNA4mC method for *A. thaliana*, *C. elegans*, and *D. melanogaster*, respectively.

We finally evaluate the global performance of DeepDNA4mC with receiver operating characteristic (ROC) and precision-recall (PR) curves. These curves are typical of an accurate classifier, and we calculated the area under these curves (AUC) based on the trapezoidal rule. When employing the DeepDNA4mC on the Zeng_*2020_1* dataset, the performance of our method showed promising results for the three species (Figure 2). The average AUC values of the ROC curves ranged from 0.920 to 0.953, and the PR curves from 0.916 to 0.946.

**FIGURE 3:**
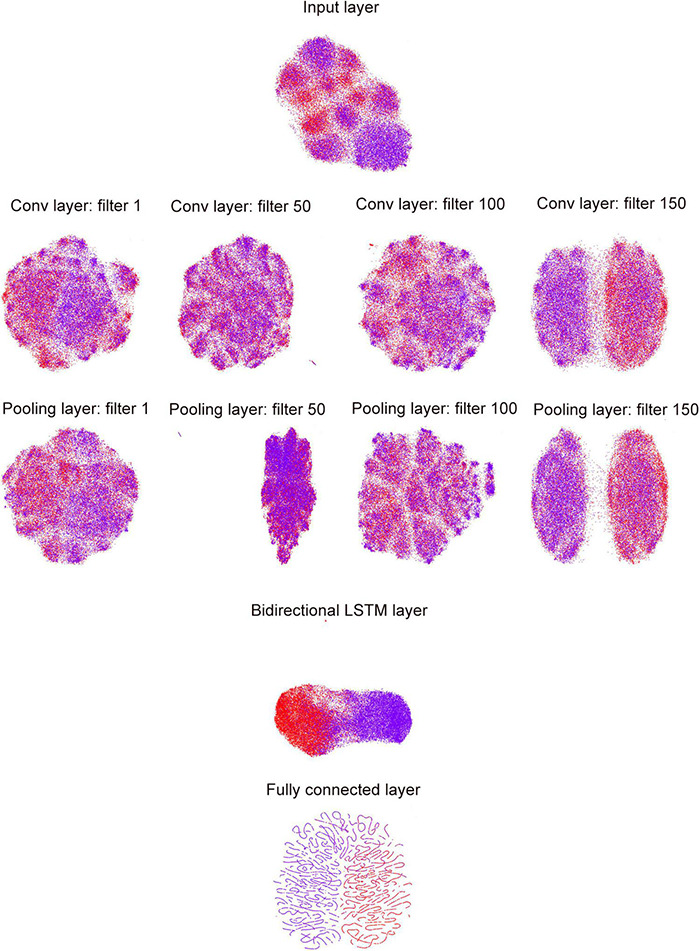
UMAP visualization of inter-layer evolution in the DeepDNA4mC for the *D. melanogaster*. Here we show the model’s internal representation of 4mC and non-4mC classes by applying layerUMAP [38], a tool for visualizing and analyzing deep learning models, to five layer representations in the DeepDNA4mC. Colored point clouds represent the different sample categories, showing how the inter-layer clusters the samples.

To investigate the ability of DeepDNA4mC to distinguish methylated and non-methylated sites, we analyzed features extracted from different network layers of the classification model and further compressed the extracted features to a 2D space using a uniform manifold approximation and projection (UMAP). UMAP is a non-linear dimensionality reduction method that maps similar input vector to close points in the reduced space and keeps the distance between clusters from the original, higher-dimensional space. First, the internal representation learned in the last fully connected layer clearly separated the positive and negative samples into two distinct clusters (Figure 3). The positive samples were mainly clustered on the left, in contrast to the negative samples, which were clustered on the right. Second, the inter-layer evolution can reflect the changes in internal representation as the observations “flow through” the layers of the network. For example, the representations of sample become more and more discriminative along the layer hierarchy, with them mixed in the input layer, culminating with a clear separation in the last fully connected layer. The deeper the layer level, the better the separation, as observed in Figure 3. Third, we could employ the same idea to visualize inter-filter evolution in the convolutional and pooling layers. In this case, we can observe which filter can extract the most effective features from the input sequences, e.g., the filter 150 separated all samples into distinct clusters more clearly than the other three filters. Using UMAP can make the visualization possible since the features have been projected into 2D space by the manifold learning methods. However, the exact learned features from these filters have multiple-dimension, which cannot be plotted in to a 2D-plot. Thus, in this work, the UMAP visualization was only used for demonstrating the separation of samples from the hidden layers. The UMAP visualization of the last hidden layer representations of other deep learning models (CNN and RNN) is shown in Supplementary Figure 1.

**FIGURE 4:**
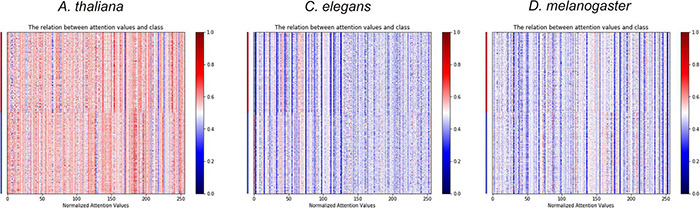
The heat maps show the importance of hidden neurons in the BiLSTM layer of DeepDNA4mC on the classification of 4mC and non-4mC sites.

As shown in Table 5, adding the self-attention layer after the BiLSTM layer of the CNN-RNN architecture can further improve the performance of the model. Furthermore, the heat map of attention values and sample classes can visualize the importance of each hidden neurons in the BiLSTM layer of different deep learning architectures for classification (Figure 4 and Supplementary Figure 2).

**FIGURE 5:**
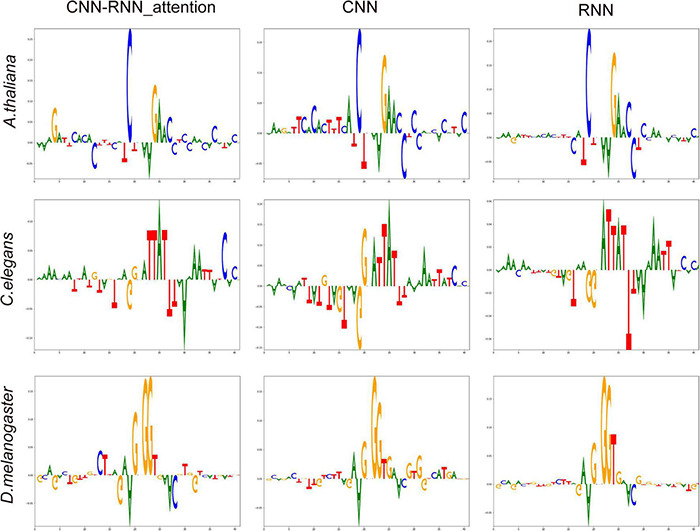
The most important features at each position of input sequence associated with N^4^-methylcytosine (4mC) determined by Deep SHAP (deep learning models). On the summary sequence logo plot, the position of the nucleotide on the *x*-axis shows its SHAP value. Positive and negative SHAP values are linked with methylation and non-methylation, respectively. The height of the logo indicates the SHAP value of the relevant nucleotide for that particular input sequence.

To evaluate each of the four nucleotides at every position of input sequence associated with 4mC site identification in a more systematic manner, we performed Deep SHAP (SHapley Additive exPlanations merged into deep learning algorithms) using 32,000 training sequences with the one-hot encoding. When high SHAP values were linked with 4mC and non-4mC sites, then the corresponding nucleotides were classified as favored and disfavored features, respectively. The most important feature (the highest SHAP value) at each position of input sequence was displayed in a sequence logo representation (Figure 5). We observed that the central and downstream regions [(−2 bp, +6 bp)] played the most important role in predicting 4mC across different species; however, three different patterns of DNA motifs were revealed within these regions, indicating a diversity of 4mC *cis*-regulatory patterns. The most important features of DeepDNA4mC (CNN-RNN_attention) for each specie were as follows: for *A. thaliana*, CGAAC, [position: (−2 bp, +3 bp, +4 bp, +5 bp, +6 bp), SHAP values: (0.275, 0.139, 0.105, 0.042, 0.062)]; for *C. elegans*, ATTAT, [position: (+1 bp, +2 bp, +3 bp, +4 bp, +5 bp), SHAP values: (0.034, 0.084, 0.085, 0.137, 0.085)]; for *D. melanogaster*, GGGT, [position: (−1 bp, +1 bp, +2 bp, + 3bp), SHAP values: (0.113, 0.172, 0.177, 0.036)].

We also noticed that even though different deep learning models exhibited different SHAP distributions, the same important features were identified by all models, for example, the CGAAC of *A. thaliana*, as shown in Figure 5.

### Performance Assessment on the *Zeng_2020_2* Dataset

Like the previous benchmark, for each deep learning architecture, the hyperparameters were tuned on the validation data and reported performance was evaluated on the held-out test set. As shown in Supplementary Table 4, unlike the benchmark results for the Zeng_*2020_1* dataset, we found that the CNN achieved the best performance for *C. elegans* in the Zeng_*2020_2* dataset. With the optimal hyperparameters, the CNN achieved an average *ACC* of 91.6% and an average *MCC* of 0.821. Its performance was followed by the CNN-RNN (90.5% and 0.796) and RNN (88.1% and 0.746). We also employed the attention mechanism in the CNN-RNN and RNN architectures to further improve their prediction performance. Our experimental results showed that applying attention mechanism *via* adding self-attention layer led to an improvement for predicting 4mC sites over the results without applying attention mechanism (Supplementary Table 5). The test *MCC* increased from 0.746 to 0.783 and 0.796 to 0.808 for RNN and CNN-RNN models, respectively. However, the improved performance was still slightly weaker than that of the CNN model. In addition to one-hot encoding, we finally assessed whether other encoding methods improved the performance the CNN model in the 4mC prediction task. Consistent with previous results, 1-mer_onehot again achieved the best performance and other methods did not improve the performance of CNN on the Zeng_*2020_2* dataset (Supplementary Table 6).

## Conclusion

DNA chemical modifications can influence biological function. In prokaryotic and eukaryotic genomes, methylation of DNA base yields several modification types: C^5^-methylcytosine (5mC), N^6^-methyladenine (6mA), and N^4^-methylcytosine (4mC). These marks play a role in gene regulation, imprinting, aging and disease. Compared to the studies on 5mC and 6mA, the 4mC has received more attention for its many important and unknown biological functions. Inferring the methylation status of individual N^4^-cytosine in a genome is the first task to elucidate the regulatory mechanisms of 4mC and benefit the in-depth exploration of its functional effects. Several methods can detect N^4^-methylcytosine, and among these methods, deep learning has been at the forefront of 4mC identification in recent years, thus becoming the focus of this study. So far, at least 11 deep learning-based methods have been developed to predict potential DNA 4mC sites from sequence at single-nucleotide resolution (Table 1). We hope the summary of these state-of-the-art methods, the detailed strategy descriptions, and the recommendations and guidelines for choosing training datasets, deep learning architectures, encoding and validation methods, and web servers can assist researchers in the development of their own models.

We have proposed a set of methodologies based on deep learning techniques to develop better predictors. In two benchmark studies, we have primarily focused on three types of deep learning architectures: convolutional neural networks (CNNs), recurrent neural networks (RNNs), and convolutional-recurrent neural networks (CNN-RNNs). We have systematically analyzed several important factors, such as model architecture and its hyperparameters (the number of filters, kernel, pooling, and BiLSTM sizes, etc.), encoding methods and attention mechanisms, in order to assess their contribution to 4mC prediction. In our analysis, we observed large differences in the performance among the methods in response to changing above factors. In addition, incorporating the attention mechanisms in the RNN and CNN-RNN architectures improved the performance. Based the first benchmark result, we recommend the use of the hybrid CNN-RNN_attention model (with one-hot encoding) since it had a better performance compared to the other models or methods tested across three representative model organisms. The proposed CNN-RNN_attention model (DeepDNA4mC) can identify 4mC sites at single-nucleotide resolution with high accuracy and reliability. For the second benchmark dataset, the CNN stood out among the three deep learning architectures and performed best in terms of the standard metrics of accuracy and *MCC* score. The CNN-RNN_attention performed slightly worse than CNN, which suggests that there is no single winner among the three algorithms. Taken together, researchers should design suitable models or methods for specific 4mC data, and choose the right deep learning architecture with the right encoding method to develop better predictors.

We have also introduced several novel visualization techniques in an attempt to better analyze and understand deep learning models. First, we have shown that the UMAP can be used to visualize the relationships between learned representations of 4mC and non-4mC samples. Through experiments conducted in the *D. melanogaster* dataset, we have shown how to visually track inter-layer and inter-filter evolution of learned representations. The UMAP can give insight into the function of intermediate feature layers and provide valuable visual feed-back for network designers. We next attempted to identify sequence features associated with 4mC sites using the Deep SHAP method. The most important features of each position of input sequence were visualized by a sequence logo plot. Deep SHAP can uncover a few interesting regulatory patterns that cannot be detected by conventional motif analysis. These new motif patterns based on SHAP values are worthy of further investigation in epigenetics.

## Data Availability Statement

The original contributions presented in the study are included in the article/Supplementary Material, further inquiries can be directed to the corresponding author/s.

## Author Contributions

JL and LY conceived the study and wrote the manuscript. RJ and YZ contributed to the design, implementation, and testing of the model. QC and FL performed the data analysis. All authors read and agreed to the published version of the manuscript.

## Conflict of Interest

The authors declare that the research was conducted in the absence of any commercial or financial relationships that could be construed as a potential conflict of interest.

## Publisher’s Note

All claims expressed in this article are solely those of the authors and do not necessarily represent those of their affiliated organizations, or those of the publisher, the editors and the reviewers. Any product that may be evaluated in this article, or claim that may be made by its manufacturer, is not guaranteed or endorsed by the publisher.
