# Supplementary material for: Systematic Analysis and Accurate Identification of DNA N4-Methylcytosine Sites by Deep Learning
Source: Front Microbiol. 2022 Mar 15;13:843425. doi: 10.3389/fmicb.2022.843425 (PMC8989013; doi:10.3389/fmicb.2022.843425)
Supplement: Supplementary file 1 [file Data_Sheet_1.docx]

Supplementary Material

# Brief Description of existing deep learning-based algorithms and implementations for 4mC prediction

We reviewed the existing deep learning algorithms and implementations and described their deep learning architectures, feature encoding methods, training datasets, species, web servers and validation methods (**Table 1**). As described earlier, a total of 14 deep learning-based computational approaches have been developed for DNA 4mC site prediction, which are called 4mCCNN, 4mcDeep-CBI, Deep4mcPred, Deep4mC, DeepTorrent, DNC4mC-Deep, mCNLP-Deep, 4mCPred-CNN, 4mC-w2vec, iRG-4mC, 4mCPred-MTL, i4mC-Deep, Deep-4mCW2V, and DCNN-4mC respectively (Khanal et al., 2019; Zeng et al., 2020; Zeng and Liao, 2020; Xu et al., 2021; Liu et al., 2021; Wahab et al., 2020; Wahab et al., 2021; Abbas et al., 2021; Khanal et al., 2021; Lim et al., 2021; Zeng et al., 2021; Alam et al., 2021; Zulfiqar et al., 2021; Rehman et al., 2021). A more detailed description of 11 computational approaches is also provided below in chronological order.

## 4mCCNN

As the first deep learning-based classifier for identifying 4mC sites, 4mCCNN was built by Khanal *et al*. in 2019 based on the CNN and one-hot encoding method (Khanal et al., 2019). Based on the datasets established by Chen *et al*. (Chen et al., 2017) and parameter optimization during training, the algorithm was designed to recognize 4mC sites in six species, including *A. thaliana*, *C. elegans*, *D. melanogaster*, *E. coli*, *G. pickeringii*, and *G. subterraneus**.* Using the 10-fold cross validation on the benchmark datasets, the predictive performance of 4mCCNN outperformed other exiting methods (e.g., iDNA4mC, 4mCPred and 4mcPred-SVM) in all the six species. A web sever of 4mCCNN is freely available at <https://home.jbnu.ac.kr/NSCL/4mCCNN.htm.>

## 4mcDeep-CBI

4mcDeep-CBI was developed by Zeng *et al*. based on a hybrid deep learning model (CNN-BiLSTM) and eight sequence-based features, such as BKF, DBPF, KNN, PCP, MMI, PseDNC, PseEIIP, and RFHCP (Zeng et al., 2020). After rigorous data processing, they created a new dataset consisting of 11,173 positive samples and 6,635 negative samples by searching the genomic sequences of *C*. *elegans* from the MethSMRT database (Ye et al., 2016). Unlike other studies, the 3-fold cross validation rather than 10-fold cross validation was chosen to assess the prediction models. Compared with another predictor, 4mCpred-IFL (a typical SVM-based predictor), 4mcDeep-CBI not only obtained better prediction performance but also ran much faster. A standalone version of 4mcDeep-CBI is freely accessible at <https://github.com/mat310/4mcDeep.>

## Deep4mcPred

Deep4mcPred was proposed based on a multi-layer deep learning network, which integrated the residual network (ResNet), RNN, and the attention mechanism (Zeng and Liao, 2020). Zeng and Liao also used one-hot encoding method to characterize DNA sequences. According to the data processing procedure proposed by Chen *et al*. (Chen et al., 2017), they reconstructed a larger and more balanced dataset with samples from three species: *A. thaliana*, *C. elegans*, and *D. melanogaster*. Using the 10-fold cross validation on this dataset, the performance of Deep4mcPred was superior to that of other four exiting SVM-based methods (iDNA4mC, 4mCPred, 4mcPred-SVM and 4mcPred-IFL). Further studies have shown that the attention mechanism is indeed useful for acquiring discriminative feature representations. A web sever of Deep4mcPred is publicly accessible at [http://server.malab.cn/Deep4mcPred.](https://home.jbnu.ac.kr/NSCL/4mCCNN.htm.)

## Deep4mC

Deep4mC was established on a CNN model with the attention mechanism (Xu et al., 2021). Based on a systematic analysis of the contribution of different features to the model performance, Xu *et al*. selected binary, ENAC, EIIP, and NCP as representative features to encode the DNA sequences. Two benchmark datasets from six species (*A. thaliana*, *C. elegans*, *D. melanogaster*, *E. coli*, *G. pickeringii*, and *G. subterraneus*) were used for model training and comparisons, one of which was primitively constructed by Chen *et al.* (Chen et al., 2017), and the other was reconstructed from the MethSMRT database for the same six species (Ye et al., 2016). Using the n-fold cross validation (n = 4, 6, 8, 10) on above datasets, Deep4mC afforded an average AUC value higher than 0.9 for all species and achieved better predictive performance compared to another method (Meta-4mCpred). A web sever of Deep4mC is publicly available at <https://bioinfo.uth.edu/Deep4mC>.

## DeepTorrent

DeepTorrent was proposed based on a hybrid deep learning model, which consisted of the CNN, BiLSTM and attention mechanism (Liu et al., 2021). Liu *et al*. used four different encoding schemes to represent the DNA sequences, which are different combinations of seven features, including one-hot encoding, CKSNAP, NCP, EIIP, ENAC, ANF, and PSTNPss. Two different datasets containing six species (*A. thaliana*, *C. elegans*, *D. melanogaster*, *E. coli*, *G. pickeringii*, and *G. subterraneus*) were used for training and test, one was from the study of Chen *et al*. (renamed as *Lin_2017*) (Chen et al., 2017), and the other was created by collecting the newly added 4mC sites for the six species from the MethSMRT database (Ye et al., 2016), called *Li_2020*. Performing the 10-fold cross validation on the *Lin_2017* training dataset, DeepTorrent obtained the highest values of *ACC*, *racall*, and *MCC* for all methods involved in the comparison (iDNA4mC, 4mCPred, 4mcPred-SVM, Meta-4mCPred and 4mCCNN). For the *Li_2020* training dataset, DeepTorrent achieved an average AUC value of 0.94 and an average ACC value of 0.87 for the six species. A web sever of DeepTorrent is freely available at http://DeepTorrent.erc.monash.edu/.

## DNC4mC-Deep

DNC4mC-Deep was developed based on the CNN and six encoding methods, including 2Kmer, 3Kmer, binary encoding, NCP, NCPNF, and MMI (Wahab et al., 2020). A benchmark dataset established by Hasan *et al*. was used for modeling and comparison, which covers the DNA sequences of *F. vesca* and *R. chinensis* (Hasan et al., 2020a). Furthermore, a cross-species dataset was created by using this benchmark dataset. Using the 10-fold cross validation on the benchmark dataset, DNC4mC-Deep not only obtained better outcomes for *F. vesca* and *R. chinensis* species compared to i4mC-ROSE, but also showed satisfactory performance in cross-species prediction. Wahab *et al*. first used heat maps to show the mutated modifications of variants and used saliency maps to explore the portions of the DNA sequences that have the most influence on model prediction performance. A web server of DNC4mC-Deep is freely accessible at http://home.jbnu.ac.kr/NSCL/DNC4mC-Deep.htm.

## 4mCNLP-Deep

4mCNLP-Deep was proposed by Wahab *et al*. based on the CNN and word embedding approach (Wahab et al., 2021). The benchmark dataset for *C. elegans* was acquired from the work of Zeng *et al*. (Zeng et al., 2020), which consisted of 11173 positive samples and 6635 negative samples. The parameters of CNN models were optimized by a grid search algorithm and DNA sequences were transformed into digital matrices by using 3-mer corpus word2vec. Performing a 3-fold cross-validation on the benchmark dataset, 4mCNLP-Deep outperformed 4mCDeep-CBI with 1.1%, 0.6%, 0.58%, 0.77%, and 4.89% improvements in *ACC*, *MCC*, *Sp*, *Sn*, and *AUC*, respectively. Like their previous study of the DNC4mC-Deep, they used heat maps and saliency maps to understand and analyze the final model. A web server of 4mCNLP-Deep is freely available at http://nsclbio.jbnu.ac.kr/tools/4mCNLP-Deep/.

## 4mCPred-CNN

4mCPred-CNN was constructed by Abbas *et al*. based on the CNN and one-hot encoding method (Abbas et al., 2021). To develop the 4mCPred-CNN and compare it with the two existing methods, a standard dataset of *Mus musculus* (*M. musculus*) used for 4mCpred-EL (Manavalan et al., 2019a) and i4mC-Mouse (Hasan et al., 2020b) was used, which was further divided into a training set and an independent set. All DNA sequences were encoded by one-hot encoding. The 10-fold cross validation of the training and independent sets indicated that the 4mCPred-CNN outperformed 4mCpred-EL and i4mC-Mouse on *ACC*, *MCC*, *Sp*, *Sn*, and *AUC*. They further investigated the effectiveness of proposed model in the case of DNA motifs changes by mutating all possible nucleotides and provided a heat map to estimate the effect of mutations. A web server of 4mCNLP-Deep is publicly available at <http://nsclbio.jbnu.ac.kr/tools/4mCPred-CNN/.>

## 4mC-w2vec and iRG-4mC

Both 4mC-w2vec (Khanal et al., 2021) and iRG-4mC (Lim et al., 2021) were proposed by Kil To Chong team based on the CNN to identify DNA 4mC sites in *Rosaceae* genome. The former employed word2vec to encode DNA sequences with a slightly small sample size, while the latter utilized the one-hot encoding and NCP (Nucleotide chemical property) to transform DNA sequences into digital vectors with a slightly larger sample size. Investigated using the independent dataset, 4mC-w2vec can also handle balanced and imbalanced blind datasets with better predictive performance than i4mC-Fuse and DNC4mC-Deep. The overall prediction performance of iRG-4mC was significantly superior to that of i4mC-ROSE when 10-fold cross validation was performed on the training and independent sets. The web servers of 4mC-w2vec and iRG-4mC are freely available at <http://nsclbio.jbnu.ac.kr/tools/4mC-w2vec/> and [http://nsclbio.jbnu.ac.kr/tools/iRG-4mC/](http://nsclbio.jbnu.ac.kr/tools/iRG-4mC/.), respectively.

**1.10 4mCPred-MTL**

4mCPred-MTL was developed by using multi-task learning and Transformer, where DNA sequences were converted into feature matrices by one-hot encoding (Zeng et al., 2021). The same dataset (Zeng*_2020_1*) established by Zeng *et al.* (Zeng and Liao, 2020) was used again, but randomly separated into the training and test datasets in a ratio of 8:2. Using the 10-fold cross validation for a fair comparison, 4mCPred-MTL outperformed other three existing methods (4mcPred-IFL, 4mcPred_SVM, and Deep4mcPred). Experimental results suggested that multi-task learning can better capture the features of DNA 4mC sites compared to the existing common features. There is no web server provided for 4mCPred-MTL.

# Supplementary Figures and Tables

**Supplementary Table 1.** **Summary of currently available** **DNA 4mC** **site prediction tools and** **their corresponding datasets**

| Year | Tools | Algorithms | Training Datasets | Encoding methods | URL | Evaluation strategy | Species | Ref |
| --- | --- | --- | --- | --- | --- | --- | --- | --- |
| 2017 | iDNA4mC | SVM | Chen *et al*’s dataset | RFHCP | yes | LOOCV | *A. thaliana*,  *C. elegans*,  *D. melanogaster*, *E. coli*,  *G. pickeringii*,  *G. subterraneus* | (Chen et al., 2017) |
| 2018 | 4mCPred | SVM | Chen *et al*’s dataset | PSTNP, EIIP | yes | LOOCV | *A. thaliana*,  *C. elegans*,  *D. melanogaster*, *E. coli*,  *G. pickeringii*,  *G. subterraneus* | (He et al., 2019) |
|  | 4mcPred-SVM | SVM | Chen *et al*’s dataset | Kmer, MBE, DBE, LPDF | yes | 10-fold CV | *A. thaliana*,  *C. elegans*,  *D. melanogaster*, *E. coli*,  *G. pickeringii*,  *G. subterraneus* | (Wei et al., 2019a) |
| 2019 | Meta-4mCpred | RF, ERT, GB, SVM | Chen *et al*’s dataset | Kmer, MBE, DPE, LPDF, RFHCP, DPCP, TPCP | yes | 10-fold CV | *A. thaliana*,  *C. elegans*,  *D. melanogaster*, *E. coli*,  *G. pickeringii*,  *G. subterraneus* | (Manavalan et al., 2019b) |
|  | 4mcPred-IFL | SVM | Chen *et al*’s dataset | Kmer+MBE, DBE+LPDF, PCPs, PseDNC, KNN, EIIP, MMI, RFHCP | yes | 10-fold CV | *A. thaliana*,  *C. elegans*,  *D. melanogaster*, *E. coli*,  *G. pickeringii*,  *G. subterraneus* | (Wei et al., 2019b) |
|  | DNA4mcEL | Ensemble | Chen *et al*’s dataset | Sequence characteristics， physicochemical properties and evolutional derivatives | yes | 10-fold CV | *A. thaliana*,  *C. elegans*,  *D. melanogaster*, *E. coli*,  *G. pickeringii*,  *G. subterraneus* | (Gong et al., 2019) |
|  | 4mCCNN | CNN | Chen *et al*’s dataset | MBE | yes | 10-fold CV | *A. thaliana*,  *C. elegans*,  *D. melanogaster*, *E. coli*,  *G. pickeringii*,  *G. subterraneus* | (Khanal et al., 2019) |
|  | 4mCpred-EL | Ensemble | 800 4mCs and 800 non-4mCs | Kmer, DPE+LPDF, RFHC, EIIP, MBE, DPCP, TPCP | yes | 10-fold CV | *M. musculus* | (Manavalan et al., 2019a) |
|  | i4mC-ROSE | RF | *F. vesca* (4854 4mCs and 4854 non-4mCs), *R. chinensis* (2337 4mCs and 2337 non-4mCs) | KSNC, MBE, EIIP | yes | 10-fold CV | *F. Vesca*,  *R. chinensis* | (Hasan et al., 2020a) |
| 2020 | iEC4mC-SVM | SVM | 388 4mCs and 388 non-4mCs | MBE, RFHC, DAE, X-k-YCF, Kmer | – | 10-fold CV | *E. coli* | (Lv et al., 2020b) |
|  | DNA4mC-LIP | Ensemble | Chen *et al*’s dataset | integration of six existing predictors | yes | independent evaluation | *A. thaliana*,  *C. elegans*,  *D. melanogaster*, *E. coli*,  *G. pickeringii*,  *G. subterraneus* | (Tang et al., 2020) |
|  | 4mcDeep-CBI | CNN, BLSTM | *Zeng_2020**_2* dataset (11173 4mCs and 6635 non-4mCs) | Kmer+MBE, DBE+LPDF, PCPs, PseDNC, KNN, EIIP, MMI, RFHCP | – | 3-fold CV | *C. elegans* | (Zeng et al., 2020) |
|  | iDNA-MS | RF | 7899 samples 4mCs and 7898 non-4mCs for all species | Kmer, RFHCP, MBE | yes | 5-fold CV | *C. equisetifolia*,  *F. vesca*,  *S. cerevisiae*,  *Ts. SUP5-1* | (Lv et al., 2020a) |
|  | i4mC-Mouse | RF | 746 4mCs and 746 non-4mCs | Kmer, KSNC, MBE, EIIP | yes | 10-fold CV | *M. musculus* | (Hasan et al., 2020b) |
|  | Deep4mcPred | ResNet, RNN, attention mechanism | *Zeng_2020_1* dataset | One-Hot Encoding | yes | 10-fold CV | *A. thaliana*,  *C. elegans*,  *D. melanogaster* | (Zeng and Liao, 2020) |
|  | Deep4mC | CNN, attention mechanism | 1.Chen *et al*’s dataset  2.Xu *et al*’s dataset | binary, ENAC, EIIP and NCP | yes | 10-fold CV | *A. thaliana*,  *C. elegans*,  *D. melanogaster*, *E. coli*,  *G. pickeringii*,  *G. subterraneus* | (Xu et al., 2021) |
|  | DeepTorrent | CNN-RNN, attention mechanism | 1.Chen *et al*’s dataset  2.*Li_2020* dataset | cCKSNAP, NCP, EIIP, ENAC, ANF, PSTNPss | yes | 10-fold CV | *A. thaliana*,  *C. elegans*,  *D. melanogaster*, *E. coli*,  *G. pickeringii*,  *G. subterraneus* | (Liu et al., 2021) |
|  | SOMM4mC | Markov model | Chen *et al*’s dataset | The second-order Markov model | yes | 10-fold CV | *A. thaliana*,  *C. elegans*,  *D. melanogaster*, *E. coli*,  *G. pickeringii*,  *G. subterraneus* | (Yang et al., 2020) |
|  | DNC4mC-Deep | CNN | *F. Vesca* (4854 4mCs and 4854 non-4mCs), *R. Chinensis* (2337 4mCs and 2337 non-4mCs), and Cross-species (7190 4mCs and 5874 non-4mCs) | 2Kmer, 3Kmer, binary encoding, NCP, NCPNF, MMI | yes | 10-fold CV | *F. Vesca*,  *R. Chinensis*,  *Cross-species* | (Wahab et al., 2020) |
|  | Zhao’s study | SVM | Chen *et al*’s dataset | OHB, SNF, KNF, KSNPF, PseDNC | yes | 10-fold CV | *A. thaliana*,  *C. elegans*,  *D. melanogaster*, *E. coli*,  *G. pickeringii*,  *G. subterraneus* | (Zhao et al., 2020) |
| 2021 | 4mCNLP-Deep | CNN | *Zeng_2020**_2* dataset (11173 4mCs and 6635 non-4mCs) | 3-mer corpus word2vec | yes | 3-fold CV | *C. elegans* | (Wahab et al., 2021) |
|  | 4mCPred-CNN | CNN | 746 4mCs and 746 non-4mCs | one-hot encoding | yes | 10-fold CV | *M. musculus* | (Abbas et al., 2021) |
|  | 4mC-w2vec | CNN | *F. vesca* (3457 4mCs and 3457 non-4mCs), *R. chinensis* (1938 4mCs and 1938 non-4mCs) | word2vec | yes | 5-fold CV | *F. vesca*  *R. chinensis* | (Khanal et al., 2021) |
|  | iRG-4mC | CNN | *F. vesca* (4854 4mCs and 4854 non-4mCs), *R. chinensis* (2337 4mCs and 2337 non-4mCs) | One-hot encoding, NCP | yes | 10-fold CV | *F. vesca*  *R. chinensis* | (Lim et al., 2021) |
|  | i4mC-EL | Ensemble | 746 4mCs and 746 non-4mCs | Kmer, EIIP | yes | 10-fold CV | *M. musculus* | (Li et al., 2021) |
|  | 4mCPred-MTL | multi-task learning coupled with Transformer, attention mechanism | *A. thaliana* (16000 4mCs and 16000 non-4mCs), *C. elegans* (16000 4mCs and 16000 non-4mCs), *D. melanogaster* (16000 4mCs and 16000 non-4mCs) | One-hot encoding, 2-gram | – | 10-fold CV | *A. thaliana*,  *C. elegans*,  *D. melanogaster* | (Zeng et al., 2021) |
|  | model_4mc | RF | 746 4mCs and 746 non-4mCs | Kmer, ENAC, CKSNAP | yes | 5-fold CV | *M. musculus* | (Zulfiqar et al., 2021a) |
|  | i4mC-Deep | CNN | Chen *et al*’s dataset | NCP, ND | yes | 10-fold CV | *A. thaliana*,  *C. elegans*,  *D. melanogaster*, *E. coli*,  *G. pickeringii*,  *G. subterraneus* | (Alam et al., 2021) |
|  | Deep-4mCW2V | CNN | 270 4mCs and 270 non-4mCs | word2vec | yes | 10-fold CV | *E. coli* | (Zulfiqar et al., 2021b) |
|  | 4mC-RF | RF | Chen *et al*’s dataset | Statistical moments, position and composition-dependent features | yes | 10-fold CV, LOOCV | *A. thaliana*,  *C. elegans*,  *D. melanogaster*, *E. coli*,  *G. pickeringii*,  *G. subterraneus* | (Alghamdi et al., 2021) |
|  | XGB4mcPred | XGBoost | Chen *et al*’s dataset | One-hot encoding | yes | 10-fold CV | *A. thaliana*,  *C. elegans*,  *D. melanogaster*, *E. coli*,  *G. pickeringii*,  *G. subterraneus* | (Wang et al., 2021) |
|  | DCNN-4mC | CNN | Rehman *et al*’s dataset | One-hot encoding | yes | 10-fold CV | Twelve different species | (Rehman et al., 2021) |
|  | LapRSRC | LapRSRC | Chen *et al*’s dataset | PSTNP | – | 10-fold CV | *A. thaliana*,  *C. elegans*,  *D. melanogaster*, *E. coli*,  *G. pickeringii*,  *G. subterraneus* | (Ding et al., 2021) |

SVM, support vector machine; RF, random forest; ERT, extremely randomized tree; GB, gradient boosting; CNN, convolutional neural network; BiLSTM, bidirectional long short-term memory; ResNet, Residual Network; RNN, recurrent neural network; CNN-RNN, convolutional neural network and recurrent neural network; LapRSRC, Laplacian Regularized Sparse Representation based Classifier. Chen *et al*’s dataset contains *A. thaliana* (1978 4mCs and 1978 non-4mCs), *C. elegans* (1554 4mCs and 1554 non-4mCs), *D. melanogaster* (1769 4mCs and 1769 non-4mCs), *E. coli* (388 4mCs and 388 non-4mCs), *G. pickeringii* (569 4mCs and 569 non-4mCs), and *G. subterraneus* (906 4mCs and 906 non-4mCs). Zeng_2020*_1* dataset contains *A. thaliana* (20000 4mCs and 20000 non-4mCs), *C. elegans* (20000 4mCs and 20000 non-4mCs), and *D. melanogaster* (20000 4mCs and 20000 non-4mCs). Xu *et al*’s dataset contains *A. thaliana* (111927 4mCs and 111927 non-4mCs), *C. elegans* (60662 4mCs and 60662 non-4mCs), *D. melanogaster* (90333 4mCs and 90333 non-4mCs), *E. coli* (2067 4mCs and 10335 non-4mCs), *G. pickeringii* (5727 4mCs and 28635 non-4mCs), and *G. subterraneus* (15135 4mCs and 75675 non-4mCs). *Li_2020* dataset contains *A. thaliana* (63720 4mCs and 63720 non-4mCs), *C. elegans* (55729 4mCs and 55729 non-4mCs), *D. melanogaster* (53970 4mCs and 53970 non-4mCs), *E. coli* (1941 4mCs and 1941 non-4mCs), *G. pickeringii* (4514 4mCs and 4514 non-4mCs), and *G. subterraneus* (9934 4mCs and 9934 non-4mCs). RFHCP, ring-function-hydrogen-chemical properties, PSTNP, position-specific trinucleotide propensity; EIIP, Electronion interaction pseudopotential; Kmer, Kmer nucleotide frequency; MBE, mononucleotide binary encoding, DBE, dinucleotide binary encoding, LPDF, local position-specific dinucleotide frequency; DPE, dinucleotide binary profile encoding; DPCP, dinucleotide physicochemical properties; TPCP, trinucleotide physicochemical properties; PCP, physicochemical property; PseDNC, pseudo-dinucleotide composition; KNN, K-nearest neighbor; KSNC, *k*-space nucleotide composition; DAC, dinucleotide physicochemical properties autocorrelation; X-k-YCF, Xmer-kGap-Ymer composition frequency; ANF, accumulated nucleotide frequency; BKF, Binary and k-mer frequency; DBPF, Dinucleotide binary profile and frequency; MMI, Multivariate Mutual Information; PseEIIP, Electronion interaction pseudopotentials of trinucleotide; Binary, Binary encoding; ENAC, Enhanced nucleic acid composition; NCP, Nucleotide chemical property; ND, Nucleotide density; CKSNAP, Composition of *K*-spaced nucleic acid pairs; PSTNPss, Position-specific trinucleotide propensity based on single-stranded characteristics; NCPNF, Nucleotide Chemical Property and Nucleotide Frequency; LOOCV, leave-one-out cross-validation; CV, cross-validation.

**Supplementary Table 2**. **Summary of the Zeng_*2020_1* dataset for the three different species**

| Species | Positive samples | Negative samples |
| --- | --- | --- |
| *A. thaliana* | 20000 | 20000 |
| *C. elegans* | 20000 | 20000 |
| *D. melanogaster* | 20000 | 20000 |

| **Supplementary Table 3. Model evaluation metrics** | |
| --- | --- |
| **Metric** | **Calculation** |
| Precision (PRE) | $PRE=\frac{TP}{TP+FP}$ |
| Accuracy (ACC) | $ACC=\frac{TP+TN}{TP+FP+TN+FN}$ |
| *F*-value | $F-value=2\times\frac{TP}{2TP+FP+FN}$ |
| Recall | $Recall=\frac{TP}{TP+FN}$ |
| Matthew’s correlation coefficient (MCC) | $MCC=\frac{\left( TP\times TN \right)-\left( FN\times FP \right)}{\sqrt{\left( TP+FN \right)\times\left( TN+FP \right)\times\left( TP+FP \right)\times\left( TN+FN \right)}}$ |
| Where *TP*, *TN*, *FP* and *FN* represent the numbers of true positives, true negatives, false positives and false negatives, respectively | |

**Supplementary Table 4. Performance comparison of different architectures on the Zeng_*2020_2* dataset**

| Model | ACC (%) | *F*-value (%) | Recall (%) | PRE (%) | MCC |
| --- | --- | --- | --- | --- | --- |
| CNN | **91.6** | **93.3** | **93.6** | **93.1** | **0.821** |
| RNN | 88.1 | 90.5 | 91.4 | 89.8 | 0.746 |
| CNN-RNN | 90.5 | 92.4 | 92.8 | 92.0 | 0.796 |

**Supplementary Table 5. Performance comparison of RNN and CNN-RNN models with or without the attention mechanism on the Zeng_*2020_2* dataset**

| Species | Models | ACC (%) | *F*-value (%) | Recall (%) | PRE (%) | MCC |
| --- | --- | --- | --- | --- | --- | --- |
| C.elegans | RNN | 88.1 | 90.5 | 91.4 | 89.8 | 0.746 |
|  | RNN_Attention | **89.9** | **91.9** | **92.5** | **91.5** | **0.783** |
|  | CNN-RNN | 90.5 | 92.4 | 92.8 | 92.0 | 0.796 |
|  | CNN-RNN_Attention | **90.9** | **92.7** | **93.0** | **92.5** | **0.808** |

**Supplementary Table 6.** Performance comparison of the CNN model with different encoding methods on the Zeng_*2020_2* dataset

| Species | Encoding | ACC (%) | *F*-value (%) | Recall (%) | PRE (%) | MCC |
| --- | --- | --- | --- | --- | --- | --- |
| *C.elegans* | 1-mer_onehot | **91.6** | **93.3** | **93.6** | **93.1** | **0.821** |
|  | 2-mer_onehot | 90.5 | 92.5 | 92.6 | 92.5 | 0.795 |
|  | 3-mer_onehot | 89.4 | 91.5 | 90.5 | 92.5 | 0.774 |
|  | 1-mer_dict | 91.4 | 93.2 | 93.9 | 92.6 | 0.815 |
|  | 2-mer_dict | 90.9 | 92.8 | 93.2 | 92.4 | 0.804 |
|  | 3-mer_dict | 89.5 | 91.5 | 91.3 | 92.0 | 0.777 |


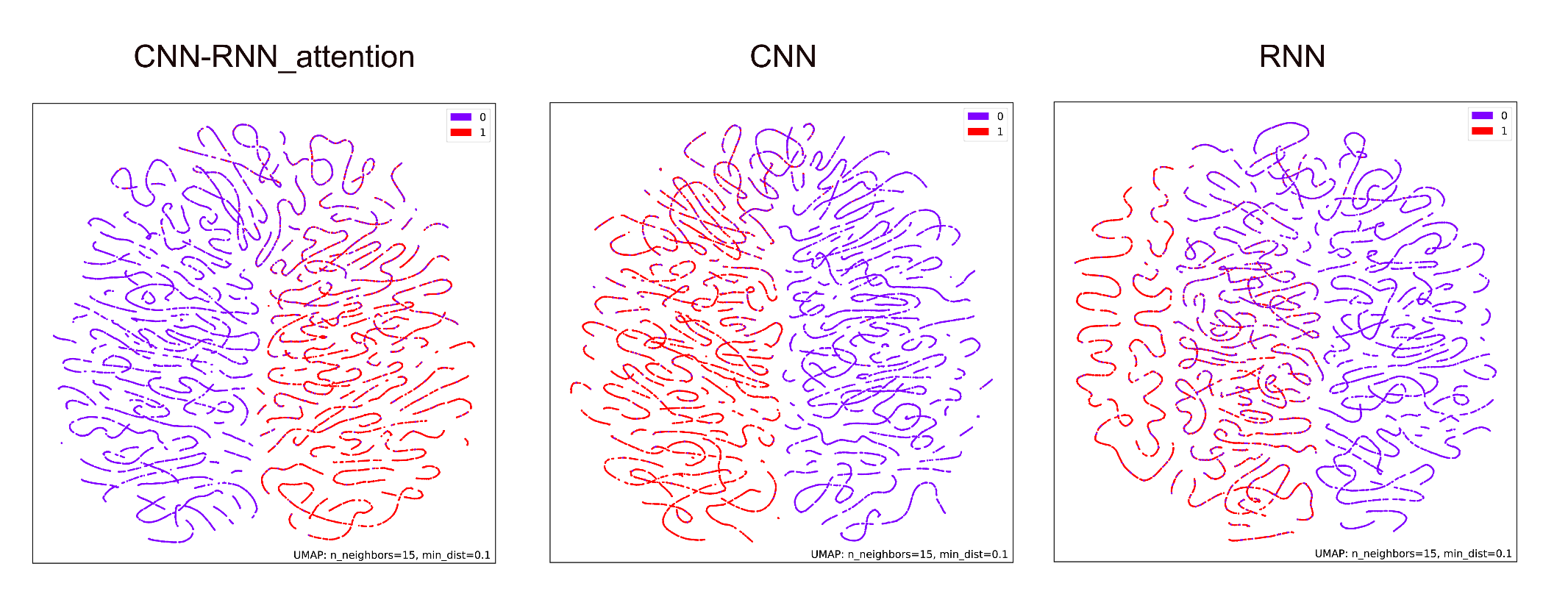


**Supplementary Figure 1. UMAP visualization of the last hidden layer representations of three deep learning models.**


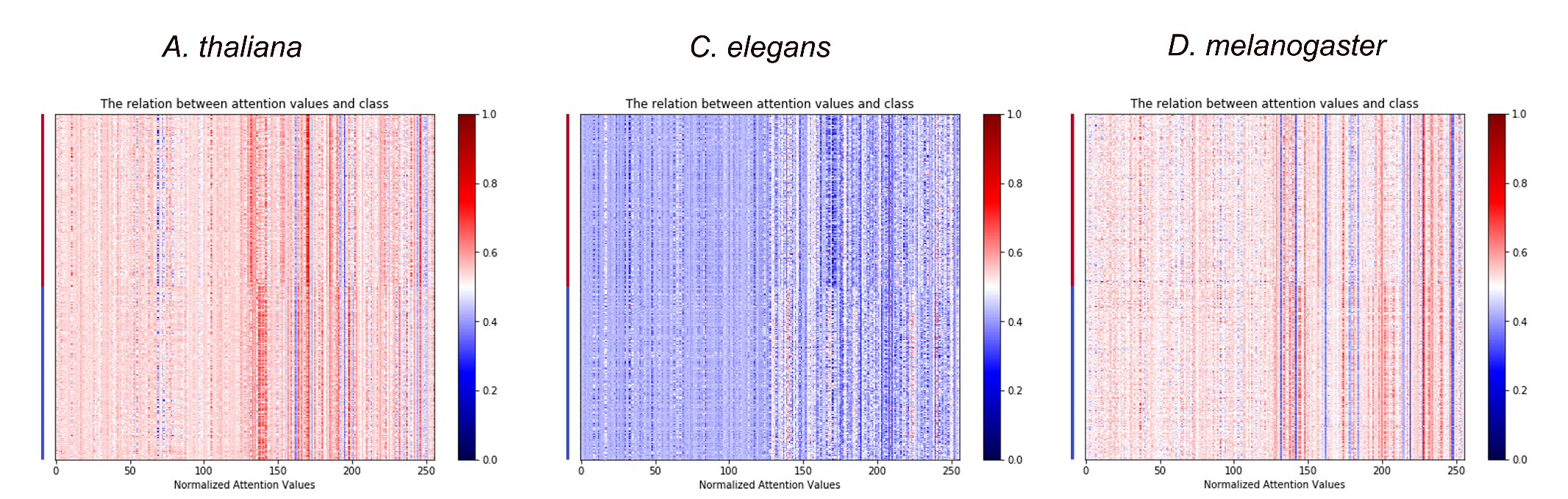


**Supplementary Figure 2. The heat maps show the importance of hidden neurons in the BiLSTM layer of RNN arechitecture on the classification of 4mC and non-4mC sites.**
